# Protection of Anthocyanin from *Myrica rubra* against Cerebral Ischemia-Reperfusion Injury via Modulation of the TLR4/NF-κB and NLRP3 Pathways

**DOI:** 10.3390/molecules23071788

**Published:** 2018-07-20

**Authors:** Hong-Xin Cui, Ji-Hong Chen, Jing-Wan Li, Fang-Rong Cheng, Ke Yuan

**Affiliations:** 1 College of Pharmacy, Henan University of Chinese Medicine, Zhengzhou 450046, China; cuihongxin1974@163.com (H.-X.C.); chenjihong0011@163.com (J.-H.C.); chengfangrong1963@126.com (F.-R.C.); 2 Collaborative Innovation Center for Respiratory Disease Diagnosis and Treatment & Chinese Medicine Development of Henan Province, Zhengzhou 450046, China; 3 Forestry and biotechnology College, Zhejiang Agriculture and Forestry University, Lin’an 311300, China; lijingwan127@126.com; 4 Jiyang College of Zhejiang Agriculture and Forestry University, Zhu’ji 311800, China

**Keywords:** *Myrica rubra*, anthocyanins, cyanidin-3-*O*-glucoside, cerebral ischemia-reperfusion, TLR4/NF-κB, NLRP3

## Abstract

*Myrica rubra* (MR) is rich in anthocyanins, and it has good anti-cancer, anti-aging, antioxidant, and antiviral effects. The proportion of disability and death caused by ischemic stroke gradually increased, becoming a major disease that is harmful to human health. However, research on effects of anthocyanin from MR on cerebral ischemia-reperfusion (I/R) injury is rare. In this study, we prepared eight purified anthocyanin extracts (PAEs) from different types of MR, and examined the amounts of total anthocyanin (TA) and cyanidin-3-*O*-glucoside (C-3-G). After one week of PAE treatment, the cerebral infarction volume, disease damage, and contents of nitric oxide and malondialdehyde were reduced, while the level of superoxide dismutase was increased in I/R mice. Altogether, our results show that Boqi^1^ MR contained the most TA (22.07%) and C-3-G (21.28%), and that PAE isolated from Dongkui MR can protect the brain from I/R injury in mice, with the mechanism possibly related to the Toll-like receptor 4 (TLR4)/ nuclear factor-κB (NF-κB) and NOD-like receptor pyrin domain-containing 3 protein (NLRP3) pathways.

## 1. Introduction

Stroke is one of the most common causes of adult disability and death worldwide, with more than 80% of all cases triggered by ischemic events. Stroke exhibits characteristics such as acute onset, unpredictability, high incidence, and presence in younger patients. Ischemic stroke typically results from thrombotic occlusion of the cerebral basilar artery (mostly the middle cerebral artery) or its branches [1,2]. The mechanism of ischemic cerebrovascular disease is complicated and multifactorial. At present, many drugs induce numerous side effects at the same time as exerting their therapeutic effect [3]. Therefore, it is vital to identify natural plant extracts that improve the current treatment status of cerebral ischemia-reperfusion (I/R) injury, to identify molecular biological mechanisms, and to investigate more effective treatment methods.

**Figure 1:**
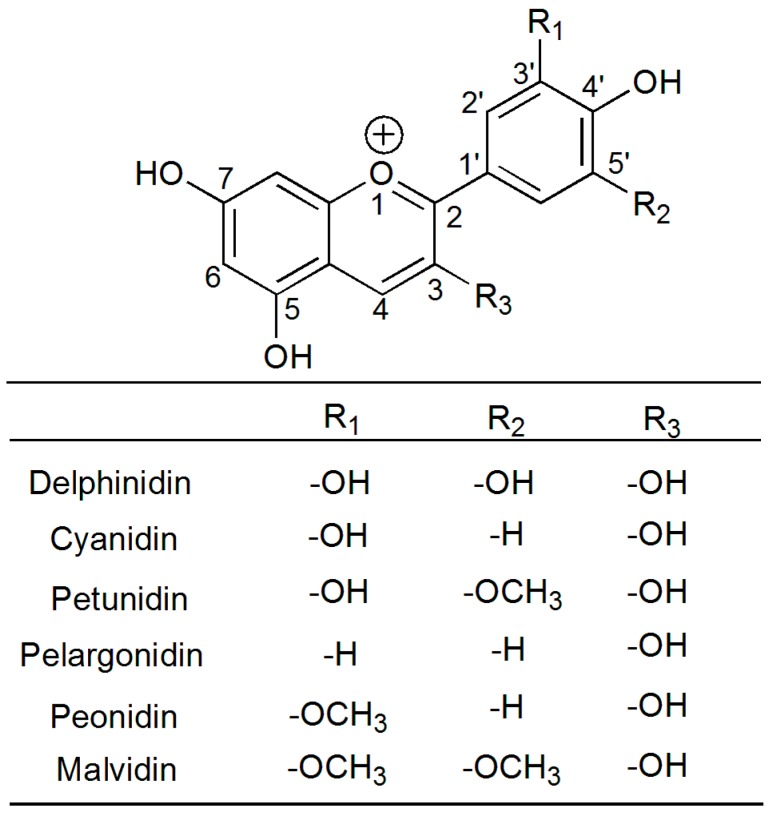
Structure of anthocyanidins.

The Myricaceae plant, *Myrica rubra* Sieb. et Zucc., has a long cultivation history and a high yield in China, and is one of the most important edible fruits with a high anthocyanin content. Anthocyanins are prominent members of the secondary plant metabolite class of flavonoid compounds, which belong to the superfamily of antioxidants named phenolics or polyphenolics [4]. Anthocyanins are derivatives of the 2-phenyl-cationic structure, with a typical flavone structure formed from the combination of glycosidic bonds with anthocyanin. Along with chlorophyll and carotenoid, they are known as the three major natural pigments, and can be divided into six main anthocyanin groups according to their substituent groups (Figure 1). Among them, cyanidin-3-*O*-glucoside (C-3-G) is one of the most common anthocyanins, and accounts for >95% of the total anthocyanin (TA) in *Myrica rubra* (MR) [5]. Many researchers showed that anthocyanins are beneficial, demonstrating antioxidant, anti-fatigue, anti-inflammatory, anti-carcinogenic, and anti-angiogenic properties [6,7,8,9,10]. The beneficial effects of anthocyanin on cerebral ischemia are well established. However, there are very few reports about MR’s effects on cerebral I/R injury. Furthermore, the related mechanisms of the Toll-like receptor 4 (TLR4)/nuclear factor-κB (NF-κB) and NOD-like receptor pyrin domain-containing 3 protein (NLRP3) pathways are still unreported. Thus, we prepared and analyzed eight kinds of purified anthocyanin extracts (PAEs) from different varieties of MR, studied their protection and mechanism with regards to cerebral I/R injury, and provided a theoretical basis for daily life and clinical research.

## 2. Materials and Methods

### 2.1. Materials and Reagents

Eight cultivars of *Myrica rubra* were obtained from the Zhejiang Province, China in June 2016. *Myrica rubra* Sieb. et Zucc. cv. Biqi^1^, *Myrica rubra* Sieb. et Zucc. cv. Tanmei, and *Myrica rubra* Sieb. et Zucc. cv. Shuijing were obtained from Ninghai; *Myrica rubra* Sieb. et Zucc. cv. Biqi^2^ was obtained from Yuyao; *Myrica rubra* Sieb. et Zucc. cv. Dongkui was obtained from Taizhou; *Myrica rubra* Sieb. et Zucc. cv. Dingdai was obtained from Wenzhou; *Myrica rubra* Sieb. et Zucc. cv. Wandao was obtained from Zhoushan; and a wild species of *Myrica rubra* Sieb. et Zucc. was obtained from Lin’an. Fruits of each cultivar were stored at −20 °C until analysis.

Methanol, triphenyl tetrazolium chloride (TTC), and C-3-G standards were purchased from the Aladdin reagent company (Shanghai, China), while the nitric oxide (NO) kit, malondialdehyde (MDA) kit, and superoxide dismutase (SOD) kit were purchased from Nanjing Biological Science and Technology Co., Ltd. (Nanjing, China). The methanol purity was chromatography grade, and the other organic solvents and chemical reagents were analytically pure.

### 2.2. Animals

Male ICR mice weighing 16–20 g were adapted to experimental conditions at 20 ± 2 °C, a humidity of 60 ± 5%, a 12-h light/dark cycle, and ad libitum access to food and water. Mice and food were purchased from the Laboratory Animal Center of the Zhejiang Academy of Medical Sciences (Hangzhou, China; Certificate Number SCXK 2014-0001). All procedures for animal experiments were in accordance with the guidelines of Chinese animal care, which conform with the international acceptance of the use of experimental animals.

### 2.3. Preparation of PAEs

**Figure 2:**
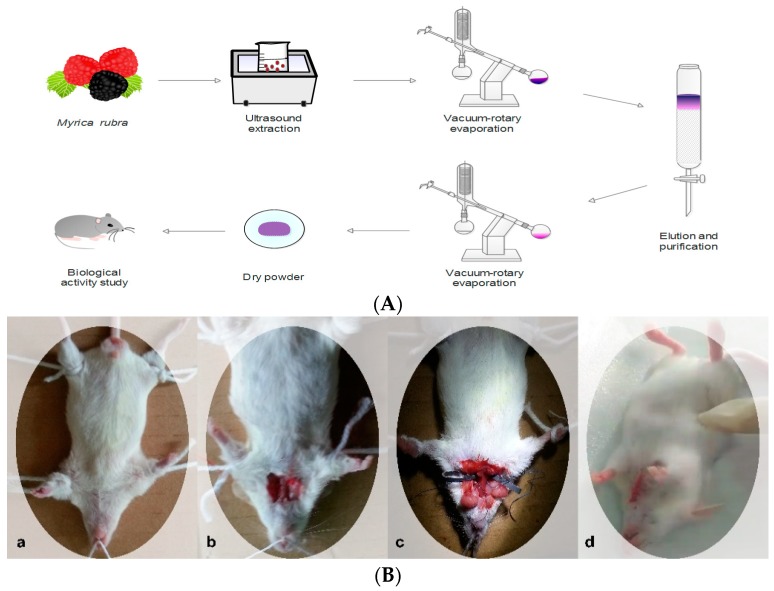
Experimental flow chart. (**A**) Process flow chart for the extraction and purification of total anthocyanin. (**B**) Experimental process of inducing cerebral ischemia-reperfusion (I/R) injury in mice: (**a**) anesthesia and fixation; (**b**) exposure of the common carotid arteries; (**c**) blocking of the blood circulation; (**d**) suture and recovery.

Approximately 500 g of MR of each cultivar was denucleated and mashed. The flesh was extracted twice with a ratio of 1:2 (*v*/*v*) at a pH 3 with a 50% ethanol solution using ultrasonic extraction for 1 h, before being subjected to a rotary evaporator under vacuum at 40 °C until the ethanol was completely evaporated. Then, it was passed through a resin column (Diaion, HP 2 MGL, Mitsubishi Co., Tokyo, Japan, 6 cm × 30 cm) depending on the adsorbent capability of each resin. C-3-G was adsorbed onto the column, while sugar, acids, and other water-soluble compounds were eluted with more than two BV (bed volume) of distilled water until the water was clear [11]. The adsorbed material was then eluted with acidified 60% ethanol (*v*/*v*, pH 3). The eluent was concentrated on a rotary evaporator at 40 °C, before the pigment powder was prepared and stored in a cool dry place until analysis (Figure 2).

### 2.4. Determination of TA in PAEs Using the pH Difference Method

A buffer solution of pH 1.0 was prepared by mixing potassium chloride (KCl) solution with 0.2 M hydrochloric acid at a ratio of 25:67. The pH value was adjusted with the KCl solution. A buffer solution of pH 4.5 was prepared by modifying the pH value of sodium acetate with hydrochloric acid. Eight types of PAE were dissolved with 50% diluted alcohol, and were fixed with the buffer solutions of pH 1.0 and pH 4.5. After incubating for 1 h in the dark, absorbance was measured using a UV-2102 PCS UV spectrophotometer (Shanghai UNICO Co., Ltd., Shanghai, China). Absorbance (A) was calculated as follows: A = (A510 nm pH 1.0 − A700 nm pH 1.0) − (A510 nm pH 4.5 − A700 nm pH4.5). The formula for TA content was, TA (*w*/*w*) = (A × M × DF × V)/(ε × L × W_t_), where M is the molecular weight, DF is the dilution factor, V is the volume, ε is the extinction coefficient, L is the optical path, and W_t_ is the fruit weight.

### 2.5. Determination of C-3-G in PAEs Using HPLC

The C-3-G content was analyzed using a Waters 2695 high-performance liquid chromatograph linked to a Waters 2996 photodiode array detector. All samples were filtered through a 0.45-μm Millipore membrane filter before injection. A 10-μL aliquot was separated using a SunFire-C18 column (250 mm × 4.6 mm, 5 μm; Waters, Milford, MA, USA) at 30 °C. The mobile phase consisted of solvent A (2% hydrochloric acid in methyl alcohol) and solvent B (water, methyl alcohol, acetonitrile, and acetic acid at a ratio of 160:90:90:40, *v*/*v*/*v*/*v*). Using an isocratic elution with the ratio of A:B as 93:7 at a flow rate of 1.0 mL/min, the C-3-G content was detected at 530 nm.

### 2.6. Grouping

All mice were randomly divided into five groups (*n* = 10) as follows: normal control (NC), treated with a saline solution and a sham operation; I/R, treated with a saline solution and cerebral ischemia-reperfusion injury; and PAE 100, PAE 150, and PAE 300, treated with 100 mg/kg, 150 mg/kg, and 300 mg/kg PAE, respectively, from Dongkui *Myrica rubra* every day, as well as cerebral ischemia-reperfusion injury. Intragastric administration was done for seven days, and all mice were free to eat during the experiment.

### 2.7. Cerebral I/R Injury

One hour after the final administration, the mice were anesthetized and fixed to a surgical platform. A neck disinfection was performed, followed by a blunt separation at the median incision to expose the right common carotid artery, external carotid artery, and internal carotid artery. The right external carotid artery was isolated and coagulated. Artery clamps were inserted into the internal carotid artery via the external carotid artery until they reached the bifurcation of the right middle cerebral artery and the anterior cerebral artery, so as to block the blood circulation in the right middle cerebral artery. The block was applied to the mice for 15 min, before the application of a 10-min perfusion; this process was repeated four times. The wounds were sutured after being blocked for up to 2 h. The mice in the normal control group underwent the operation with suturing only and no artery clipping. The mice recovered for 24 h, and blood was taken from the eyeball, while the brain tissue was rapidly removed (Figure 2).

### 2.8. Neurological Deficit Scores

Twenty-four hours after reperfusion, the neurological deficits of the mice were assessed and scored using the Bederson neurological scale [12]. The scores were recorded as follows: 0, no neurological deficit; 1, failure to fully extend left forepaw or to flex contralateral torso and forelimb; 2, reduced resistance to lateral push or circling to the left side when the mouse was held by the tail on a flat surface, despite the posture of the mouse being normal at rest; 3, spontaneous circling to the left; and 4, absence of spontaneous movement or unconsciousness.

### 2.9. Determination of Infarct Volume in Brain Tissue

The brain tissue was cut into five slices (approximately 2-mm-thick) and dyed with 2% triphenyl tetrazolium chloride (TTC) for 30 min at 37 °C. The stained brain sections were fixed with 4% paraformaldehyde. Normal brain tissue goes brick-red, while infarcted areas remain unstained [13]. The infarct volume was calculated using the Image J software (V2.1.4.7, Bethesda, Rockville, MD, USA). Cerebral infarction volume (%) = infarct volume/total section volume × 100.

### 2.10. Monitoring of NO, SOD, and MDA Levels

The brain tissue of the mice was removed and immediately homogenized. The volume of the brain tissue homogenates at a volume fraction of 10% were obtained using a ratio of 1:9 to the volume of normal saline. The contents of NO, SOD, and MDA in brain tissue were detected using the appropriate kits.

### 2.11. Histopathology

At the end of the experiment, the brain tissue was promptly excised from the animals, who were anesthetized with 4% chloral hydrate solution. The organs were rinsed with normal saline and were fixed in 10% neutral-buffered formalin for histopathological examination using hematoxylin and eosin staining.

### 2.12. Western Blotting

Total protein was extracted from the same position of the ischemic side of the mouse’s cerebral cortex using a commercial kit (Aidlab Biotechnologies Co. Ltd., Beijing, China). For Western-blot analysis, equal amounts of protein (50 μg per lane) were loaded in the wells of 12% polyacrylamide gels. After the electrophoretic run, the proteins were electrotransferred onto polyvinylidene fluoride (PVDF) membranes. Then, the membranes were incubated for 2 h in blocking buffer [1 × TBS (Tris buffer solution), 0.1% Tween 20, and 4% nonfat milk] at room temperature. All membranes were incubated in a primary-antibody solution overnight at 4 °C, and then, in a secondary-antibody solution for 2 h at room temperature. The primary-antibody dilutions were anri-TLR4 (1:500), anti-tumor necrosis factor alpha (TNF-α; 1:500), anti- nuclear erythroid 2-related factor 2 (Nrf2; 1:1000), anti-caspase-1 (1:1000), anti-NLRP3 (1:1000), and IL-18 (1:1000) (Boster Biological Technology Ltd., Wuhan, China). The membranes were washed three times for 5 min, and were incubated for 2 h at 4 °C with HRP-conjugated (horseradish peroxidase-conjugated) secondary antibodies (anti-rabbit and anti-mouse; Boster Biological Technology Ltd., Wuhan, China). The density of the bands was determined using an enhanced chemiluminescence detection system (Amersham Pharmacia, Piscataway, NJ, USA), and the gray value of the bands were quantified using the ImageJ analysis software.

### 2.13. Statistical Analysis

All data are expressed as mean ± standard deviation, and were analyzed using the SPSS statistical software (SPSS19.0 Inc., Chicago, IL, USA). One-way analysis of variance (ANOVA) with a Duncan’s test were used for inter-group comparisons. A *p*-value <0.05 was considered as statistically significant, and a *p*-value < 0.01 was considered as highly significant.

## 3. Results and Discussion

### 3.1. TA and C-3-G Contents in the Eight Varieties of MR.

MR is rich in anthocyanins, with >95% being C-3-G [5,14]. Anthocyanins are phenolic compounds, and various types of stress (e.g., ultraviolet radiation, pathogens, soil, sunlight, rainfall, genetic factors, germination, maturity, and species diversity) can influence their levels [15,16].

**Figure 3:**
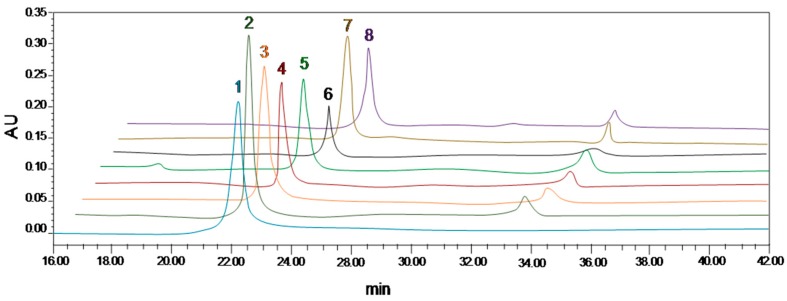
HPLC results for cyanidin-3-*O*-glucoside (C-3-G) in purified anthocyanin extracts. 1, C-3-G standard; 2, Boqi^1^; 3, Boqi^2^; 4, Wandao; 5, Dingdai; 6, Wild; 7, Dongkui; 8, Tanmei.

From Table 1 and Figure 3, it is apparent that, in addition to Shuijing, the other seven types of MR contained anthocyanins, with obvious differences in content. Among them, Boqi^1^ MR contained the most TA (22.07%) and C-3-G (21.28%), which is consistent with previous findings [5]. In general, the anthocyanin content in MR was closely related to the variety, while the same species were not affected by region.

The fruit of Dongkui MR weighs 20–25 grams, with the fruit being purple, as well as having a thicker cinnamon, soluble solid content of 13.5%, and a sugar content of 10.5%. Furthermore, it is of high and stable yield, and is wind-resistant, with mature fruits that do not fall easily. Moreover, in recent years, all localities promoted its planting. Therefore, this study investigated Dongkui MR, and examined the role of PAEs in the protection against cerebral I/R in mice, and their mechanism of action.

### 3.2. Effect of PAEs on Neurological Deficits and on Cerebral Infarct Volumes in Experimental Mice

**Figure 4:**
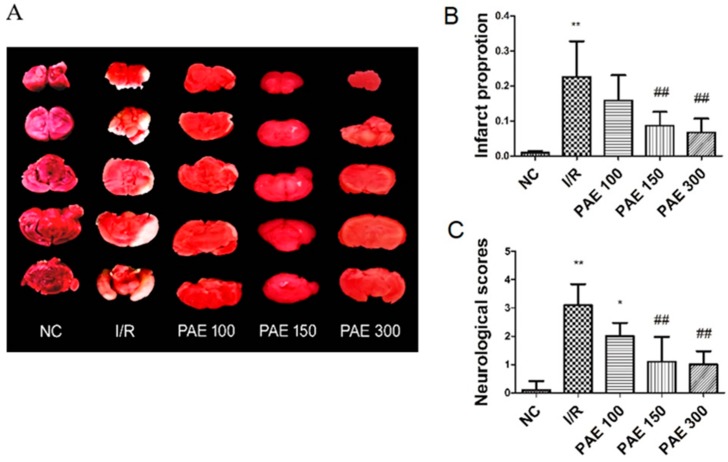
Effect of purified anthocyanin extracts (PAEs) from Dongkui *Myrica rubra* on neurological deficits and on cerebral infarct volumes in experimental mice (triphenyl tetrazolium chloride (TTC) stain, infarction remains unstained). The quantification of (**A**) is shown in (**B**); (**C**) neurological deficit scores. The data are expressed as mean ± SD (*n* = 10); * *p* < 0.05, ** *p* < 0.01 vs. negative control (NC) group; ^##^ *p* < 0.01 vs. I/R group.

Currently, ischemic cerebrovascular disease is one of the most important diseases threatening human health and survival. Reperfusion injury is also a major concern in the treatment of ischemic cerebrovascular disease. The middle cerebral artery is a vulnerable region in clinical ischemic cerebrovascular disease. Among patients with cerebral infarction, the proportion with trunk infarction of the middle cerebral artery was 82.12% [17]. Therefore, the model of middle cerebral artery I/R induced by internal carotid artery occlusion is the closest to a clinical lesion. As shown in Figure 4, the results of the evaluation of neurological function in mice showed that the brain-nerve injury in I/R mice was significantly greater than that in the NC group (*p* < 0.01). After PAE treatment, the injury was reduced, especially in the middle- and high-dose groups (*p* < 0.01). The cerebral infarct volume was detected using TTC staining. The NC group had no infarction. Compared with the NC group, the cerebral I/R injury in the I/R group was associated with obvious infarction (*p* < 0.05). Additionally, the cerebral infarct volume in mice treated with PAEs was significantly smaller when compared with that of the I/R group (*p* < 0.05), especially in the PAE 150 and PAE 300 groups (*p* < 0.01).

### 3.3. Effect of PEAs on the NO, SOD, and MDA Contents in the Brains of Experimental Mice

During cerebral ischemia, reperfusion, and recovery, multiple pathways are involved in the cascade of rapid injury, such as brain edema, excess free-radical formation, energy disruption, apoptotic gene activation, calcium overload, leukocytosis, and a series of pathological changes [18]. MDA and NO are involved during the cerebral I/R injury, while SOD is involved in preventing injury.

**Figure 5:**
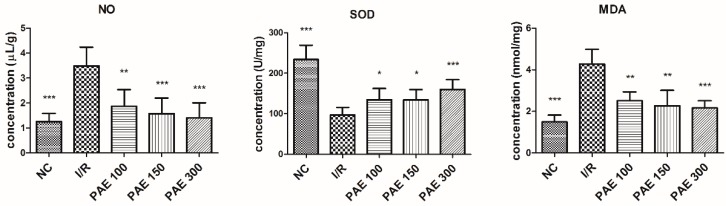
Effect of PAEs from Dongkui *Myrica rubra* on the nitric oxide (NO), malondialdehyde (MDA), and superoxide dismutase (SOD) contents in the brain tissue of mice with cerebral ischemia-reperfusion injury. The data are expressed as mean ± SD (*n* = 10); * *p* < 0.05, ** *p* < 0.01, *** *p* < 0.001 vs. I/R group.

NO is a non-traditional neurotransmitter that is produced by NO synthase, which degrades arginine. NO inhibits normal mitochondrial energy production in many pathological processes, and it has a toxic response that leads to cell death [19]. In recent years, many studies confirmed that NO plays a key role in the pathogenesis of cerebral ischemia. Therefore, NO content in the brain tissue of mice directly reflects the extent of brain damage [20]. As shown in Figure 5, compared with the NC group, NO content was significantly higher in brain tissue from the I/R group (*p* < 0.01). However, in mice treated with PAE, NO content was significantly lower in brain tissue compared with the model group (*p* < 0.01). NO plays a very complex role in cerebral ischemia, and accordingly, the results reflect the different experimental conditions.

Excess free radicals can cause lipid, protein, and nucleic-acid peroxidation. Consequently, damaged membrane structures, and irreversible cell changes and death cause microcirculatory disturbances and increased blood–brain barrier permeability in brain tissue [21]. SOD is an effective free-radical scavenger, and an important antioxidant enzyme for scavenging oxygen free radicals in the body. Thus, SOD plays a significant role in oxidation and the antioxidant balance of the organism. Decreased SOD activity results in the accumulation of a large number of free radicals in the brain. Free radicals induce lipid peroxidation of cell membranes, resulting in damage to the structure and function of cell membranes, with resultant neuronal damage. SOD’s capability as an oxygen free-radical scavenger is reduced upon increased consumption. Brain-tissue damage can lead to decreased SOD activity, increased levels of free radicals, and biofilm lipid peroxidation, resulting in large amounts of MDA. The MDA content reflects the oxygen free-radical content in the body, which reflects the extent of lipid peroxidation [20]. Our results show that PAE significantly prevents the decrease in SOD content and increase in MDA content in the brain tissue of mice (*p* < 0.01), which effectively improves cerebral I/R injury. Studies found that, after cerebral ischemia, blood flow is reconstructed with increased lipid peroxide levels in the brain and serum. Meanwhile, there is also increased tissue damage, and a greater level of lipid-peroxidation products and MDA [22].

### 3.4. Effect of PAEs on Brain Pathology in Experimental Mice

**Figure 6:**
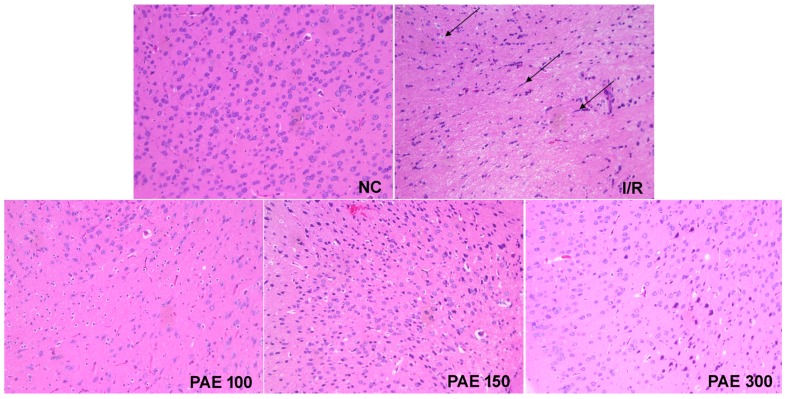
Effect of PAEs from Dongkui *Myrica rubra* on brain pathology in experimental mice (hematoxylin and eosin stain, ×200). The images are from the same region of the brain under different experimental conditions.

A pathological examination of brain tissue of mice from the NC group found normal cell structures, clear cell structures, normal cell morphologies, and clear edges, with uniform staining of the nucleus and intercellular substances. However, in mice from the I/R group, the histopathological examination revealed a sparse and disordered cell arrangement, vacuolar degeneration, cell-gap enlargement, blurring of membrane boundaries, nuclear pyknosis, contraction of cell bodies, and cellular degeneration and necrosis, as indicated by the arrows in Figure 6. Damaged brain tissue in mice treated with PAEs was not apparent, and the degree of injury in mice treated with a high dose of PAE was visibly reduced. This indicates that the extraction and purification of anthocyanin from *Myrica rubra* plays an important role in the protection of brain tissue (Figure 6).

### 3.5. Effect of PAEs on the TLR4/NF-κB Signaling Pathway

**Figure 7:**
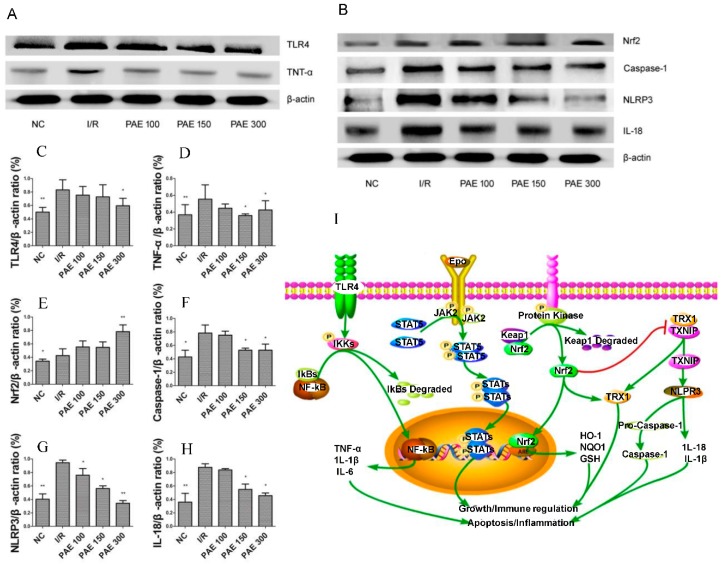
Effect of PAEs from Dongkui *Myrica rubra* on protein expression: (**A**) the expressions of Toll-like receptor 4 (TLR4), tumor necrosis factor alpha (TNF-α), and β-actin; (**B**) the expressions of nuclear erythroid 2-related factor 2 (Nrf2), caspase-1, NOD-like receptor pyrin domain-containing 3 protein (NLRP3), IL-18, and β-actin. The quantifications of (**A**) and (**B**) are shown in (**C**–**H**). All of the data are shown as mean ± SD (*n* = 10); * *p* < 0.05, ** *p* < 0.01 vs. I/R group. (**I**) The signal pathways; ↑ represents activation, while T (red line) represents inhibition.

Inflammatory reactions, apoptosis, and calcium overload are the main causes of cerebral I/R injury [23,24], with oxidative stress considered as one of the potential factors of ischemic injury. The brain needs and uses a lot of energy to function properly. With an increase in the rate of oxygen utilization, the unsaturated fatty-acid content in neural cell membranes increases, while antioxidant enzyme levels decrease, leading to vulnerability of the brain to oxidative stress [25]. A disrupted balance between oxidant and antioxidant processes is due to a breakdown of the antioxidant defense system, which is caused by the overproduction of reactive oxygen species. Reactive oxygen species destroy lipids, proteins, and nucleic acids, and alter the signaling pathways that cause cell death (necrosis or apoptosis) [25]. Therefore, antioxidants are often used as therapeutic agents for cerebral I/R injury [26]. Excitingly, anthocyanins are polyphenols that have strong antioxidant properties. As shown in Figure 7A,B, the protein expression levels of Toll-like receptor 4 (TLR4) and tumor necrosis factor α (TNF-α) in the brain tissue of mice from the I/R group were increased compared with those in that of mice from the NC group (*p* < 0.01). After PAE treatment, the expression of these proteins was reduced in the brain tissue when compared with that of the proteins in the I/R group, especially when treated with 300 mg/kg PAE (*p* < 0.05). Immunohistochemical studies show that TLR4-positive cells gradually increase in the first 22 hours after I/R [27]. TLR4 can activate nuclear factor-κB (NF-κB), ultimately causing an inflammatory response [28,29]. TLR4 also plays a key role in the pathological development of cerebral I/R injury [30], and TLR4 messenger RNAs (mRNAs) are upregulated in post-ischemic mouse brain tissue [31]. 

### 3.6. Effect of PAEs on the NLRP3 Signaling Pathway

Recent studies showed that the nuclear erythroid 2-related factor 2 (Nrf2) negatively regulates NLRP3 inflammasomes by regulating the thioredoxin-1/thioredoxin-interacting protein (TRX1/TXNIP) complex [32]. This complex inhibits caspase-1, IL-18, and IL-1β activation [33], thereby inhibiting apoptosis and inflammatory responses, protecting neurons, and reducing brain damage. Our research shows that PAE can significantly inhibit the expression of caspase-1, NLRP3, and IL-8, as well as activated Nrf2, in the brain tissue of I/R mice, thereby inhibiting inflammation and cell apoptosis, and protecting brain tissue. In addition, the regulatory effects of PAE were found to increase in a concentration-dependent manner (Figure 7).

Oxidative stress refers to the imbalance of oxidation and antioxidant activity in the body, which tends toward oxidation, causing the inflammatory infiltration of neutrophils, thereby increasing the secretion of protease and producing a large number of intermediate products of oxidation. Oxidative stress and TLR4 can activate NF-κB, thereby promoting the gene expression of various inflammatory cytokines.

Anthocyanins simultaneously attenuate inflammatory responses and oxidative stress by activating the JAK2/STAT3 signaling pathway, thereby reducing heart and cerebral I/R injury [34,35,36] (Figure 7I).

In addition, it was found that anthocyanin can inhibit the degradation of IκB induced by LPS in BV-2 cells, and the nuclear translocation of NF-κB p65 in murine neuroglia cells, thus reducing the proinflammatory mediators, including the production of nitric oxide, prostaglandin E2, and proinflammatory cytokines such as IL-1β and TNF-α [37].

Polyphenols are very important substances for improving the structure and function of capillaries and vessels, and also for helping stimulate and improve the circulatory system. Anthocyanins, which have the effect of not being oxidized by free radicals, are the only antioxidants that have the ability to protect the brain cells from the blood–brain barrier, and to protect the brain from oxidation, as well as from harmful chemicals and toxins. Studies showed that foods with a high absorbance of oxygen free radicals can increase the level of antioxidants in the blood by 10–20% [38].

## 4. Conclusions

In conclusion, of the eight cultivated types of MR, the anthocyanin content in Boqi^1^ fruit was highest (as high as 22.07%). In addition, through biochemical indices, and through macroscopic and microscopic observation of the brain tissue, we found that PAEs exert a beneficial protective effect against cerebral I/R. Furthermore, this protective effect may be related to the TLR4/NF-κB and Nrf2/antioxidant responsive element (ARE) pathways. These results may provide a theoretical basis for daily life and clinical research.

## Figures and Tables

**Table 1:** The levels of purified anthocyanin extracts (PAEs), and the total anthocyanin (TA) and cyanidin-3-*O*-glucoside (C-3-G) contents in PAEs from eight cultivars of *Myrica rubra*.

Varieties	PAE	TA	C-3-G
Boqi^1^	0.38%	22.07%	21.28%
Tanmei	0.40%	8.77%	8.59%
Shuijing	0.00%	0.00%	0.00%
Boqi^2^	0.24%	18.95%	18.57%
Dongkui	0.32%	18.10%	17.41%
Dingdai	0.38%	12.56%	11.79%
Wandao	0.35%	16.02%	15.71%
Wild	0.28%	5.10%	5.02%
